# Subconjunctival bleedings in neonatal calves: a case series report

**DOI:** 10.1186/s12917-022-03254-z

**Published:** 2022-04-27

**Authors:** Martin Steffl, Nadine Nautscher

**Affiliations:** Faculty of Agricultural Sciences, Institute of Animal Science, University of Hohenheim, Schwerzstr. 15/4, 70599 Stuttgart, Germany

**Keywords:** Subconjunctival bleeding, Anatomy, Calf, Birth, Calving difficulty, Haematology

## Abstract

**Background:**

In animals, only few reports exist about the occurrence and causes of subconjunctival bleedings, especially in newborn calves. Most case reports and studies showed that the major risk factors for subconjunctival bleedings in animals are traumatic events such as birth trauma and traffic accidents, respectively. In neonatal babies, it is suggested that compression of the thorax and abdomen during delivery or forces generated in utero during labor may raise venous pressure to conjunctival vessels and can cause subconjunctival bleedings.

**Results:**

The incidence of bleedings in neonatal Holstein–Friesian calves was 2.4 per cent of 289 neonatal calves examined over a six-year period. In general, two types of subconjunctival bleedings were seen. One was usually in a semilunar fashion immediately outside the limbus of the eye. The other type was a stripe or macule of variable size at different positions of the sclera. The subconjunctival bleedings were not related to gestational time. In all cases, affected calves were born without assistance. Multiparous cows were more often involved in the calves with subconjunctival bleedings. Two calves examined haematologically did not show signs of anemia or thrombocytopenia.

**Conclusions:**

Subconjunctival bleedings in neonatal calves appear not to be incidental findings. Main causes or associated conditions of subconjunctival bleedings were not found.

## Background

Hyposphagmas are defined as subconjunctival bleedings of the eye that can vary from dot-blot bleedings to extensive areas of bleeding that involve the entire visible sclera. They have the characteristic appearance of a sharply circumscribed redness of bleeding underneath the conjunctiva in the absence of inflammatory signs or pathological vessels [1]. Subconjunctival bleedings are usually located to the loose mounted conjunctiva bulbi. Multiple dot-blot bleedings are seen in cases of a strong connection with the episcleral tissue. The blood always has the tendency to drain into the limbus. Scleral lymphatic vessels are then responsible for the resorption [1]. This benign disorder is well described in humans and major causes include trauma and systemic vascular diseases such as hypertension [2]. However, in animals, only few reports exist about the occurrence and causes of subconjunctival bleedings. Most case reports and studies showed that the major risk factors for subconjunctival bleedings in animals are traumatic events such as birth trauma [3, 4] and traffic accidents [5]. Additionally, tumors involving the ocular adnexa may be one of the unusual causes of spontaneous subconjunctival bleedings in animals [6].

Subconjunctival bleedings subsequent to birth trauma have been documented in calves and foals [3, 4]. The incidence of subconjunctival bleedings in 169 neonatal foals examined over a four-year period was 8.3 per cent [4]. For comparison, that recorded in a study of 3573 healthy human babies revealed 1.4 per cent [7]. However, the higher incidence of subconjunctival bleedings in foals was related to foaling difficulty [4] while newborn babies in the study of Li et al. [7] showed subconjunctival bleedings after normal vaginal delivery. In this context, it is of interest that subconjunctival bleedings also occurred in newborn babies delivered by cesarean section [8]. This fact raises questions about causative factors in farm animals which could be responsible for subconjunctival bleedings in newborns.

Calving difficulties in bovine species are one of the most important topics in the veterinary practice [9]. However, to date, only one case report described subconjunctival bleeding subsequent to birth trauma in a Shorthorn calf [3].

Therefore, the purpose of this case study was to obtain more data about the occurrence and localization of subconjunctival bleedings in neonatal calves. Individual and morphological data concerning the subconjunctival bleedings in the affected calves and details of the cow’s and calf’s parturitional and gestational history were collected and analysed.

## Results

Seven of the 289 neonatal calves (2.4 per cent) had subconjunctival bleedings, involving nine eyes (1.6 per cent). Four calves showed bleedings in the left eye, one in the right eye and two calves were affected bilaterally (Table 1). All affected calves were female.

**Table 1:** Individual and morphological data concerning the subconjunctival bleedings in the seven affected calves and details of the cow’s and calf’s parturitional and gestational history

Calf	Age (days), sex	Localization	Gestational time (days)	Parity	Calving difficulty
1	1, female	B L: semilunar R: NA and T	275	1	N
2	1, female	L^a^	277	1	N
3	1, female	R: semilunar	289	3	N
4	1, female	L: D and T	282	3	N
5	1, female	L: NA and T + D	272	3	N
6	2, female	L: semilunar	270	2	N
7	1, female	B: D	280	2	N

*B* Bilateral, *L* Left, *R* Right, *D* Dorsal, *NA* Nasal, *T* Temporal, *N* no assistance

^a^no further details available

**Fig. 1:**
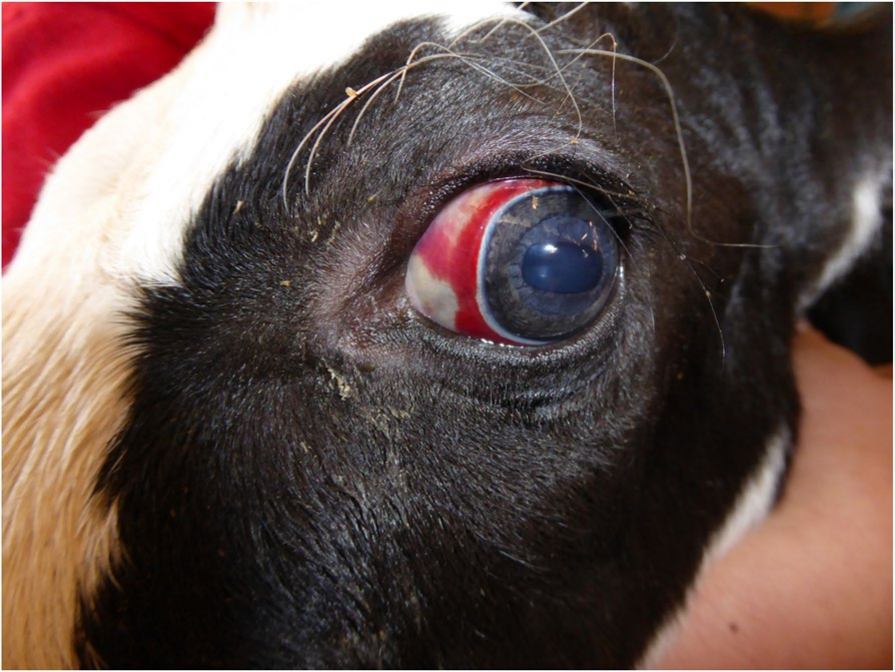
Subconjunctival bleeding in a semilunar fashion in the left eye of calf 1

**Fig. 2:**
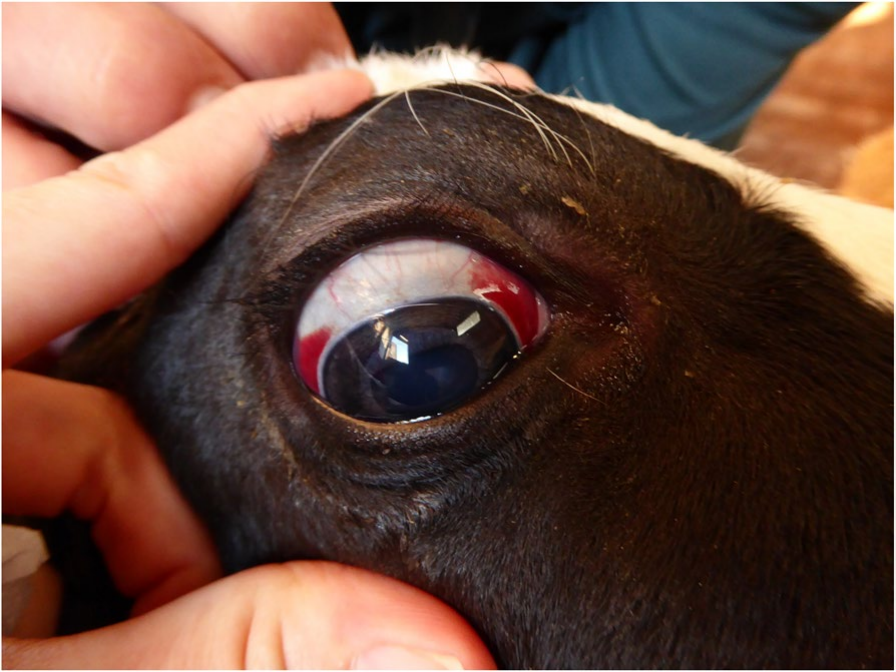
Subconjunctival bleedings extending to the limbus were located nasally and temporally (calf 1, right eye)

In general, two types of subconjunctival bleedings were seen. One was usually in a semilunar fashion immediately outside the limbus of the eye (Fig. 1). Semilunar bleedings were located nasally in dorsoventral direction or concentrated temporodorsally. The other type was a stripe or macule of variable size at different positions of the sclera (Fig. 2). Stripes or macules extending to the limbus were located nasally, temporally or dorsally and in different combinations (Table 1). In all cases, narrow well-defined blood-free stripes on the limbus were characteristic features of subconjunctival bleedings (Figs. 1 and 2). Most bleedings resolved spontaneously within 13 days of birth.

Multiparous cows were more often involved in the calves with subconjunctival bleedings (Table 1). The ratio of multiparous to primiparous cows was five (71.4 per cent) to two (28.6 per cent). There was no relationship with gestational time ranging between 270 and 289 days. In all cases, affected calves were born without assistance. Farm and/or veterinarian assistance was required on average six times per year in calves without subconjunctival bleedings.

The results of blood analyses of three affected calves are presented in Table 2. Two calves examined haematologically did not show signs of anemia or thrombocytopenia. Serum amounts of iron and/or copper were low in three calves according to reference values provided by the laboratories. Further parameters were not determined.

**Table 2:** Results of haematological and serum trace element analyses of three affected calves with subconjunctival bleedings

	Calf 1	Calf 3	Calf 7	reference interval
Leukocytes	6.0	8.7	nd	4–10 10^3^/mm^3^ [10]
Erythrocytes	7.05	7.96	nd	5–8 10^6^/mm^3^ [10]
Hemoglobin	8.7	9.2	nd	8.5–13.5 g/dl [10]
Packed cell volume	25.4	31.5	nd	28–38% [11]
MCV	36	40	nd	46–65 µm^3^ [11]
MCH	12.3	11.5	nd	11–17 pg [11]
MCHC	34.2	29.2	nd	31–34 g/dl [11]
Thrombocytes	548	488	nd	200 – 1000 10^3^/mm^3^ [10]
Neutrophil granulocytes	67	77	nd	25–45% [11]
Lymphocytes	30	17	nd	45–65% [11]
Iron	33.8^a^	nd	28^b^	100–190 µg/dl^a^ 69–249 µg/dl^b^
Copper	nd	29	33	102–203 µg/dl^b^

^a^IDEXX Vet Med Lab, ^b^Biocontrol; *nd* not determined

## Discussion

Hyposphagmas or subconjunctival bleedings (also termed hemorrhages) in newborn calves had a low incidence (2.4 per cent) in this retrospective case study. This result is in good agreement with that recorded in human babies (1.4 per cent, [7]). However, the incidence of subconjunctival bleedings in neonatal foals was 8.3 per cent [4] and thus, remarkable higher.

The higher incidence of bleedings in foals was found to be associated with different factors such as foaling difficulty, multiparity, and country of birth [4].

In our study, affected calves were born without assistance. Similarly, Li et al. [7] noted subconjunctival bleedings in neonatal babies after normal vaginal delivery. However, calving difficulties as a major factor for the development of subconjunctival bleedings in calves cannot be excluded. Calvings often occur at night and without observation by the staff. For that reason, delayed labours are often not recognized. A prolonged delivery process may cause venous pressure to conjunctival vessels, and thus cause subconjunctival bleedings.

In our study, there was the tendency that multiparous cows were more often involved in neonatal calves with subconjunctival bleedings. This finding agrees with the results by Munroe [4], but not Katzman [8]. Munroe [4] argued that the effect of multiparity in horses may be a combination of birth weight, fetal oversize, and the speed and magnitude of delivery forces in the older mare.

One surprising finding of our study was the fact that all calves with subconjunctival bleedings were female, although the ratio between male and female calves at birth was slightly biased towards the male sex. In human babies and foals, there were no statistically significant differences in the distribution of bleedings between the sexes [4, 7, 8]. Therefore, our finding that only female calves were affected should be regarded as accidental finding.

It was described that pathologies of the coagulation system, including the disorders associated with thrombocytopenia, platelet dysfunction and anemia, may cause bleeding in conjunctival vessels [2]. To gain further insights into the pathogenesis of subconjunctival bleedings we evaluated blood samples that were available from three affected calves. Two calves examined haematologically did not show signs of anemia or thrombocytopenia. Trace element deficiencies were suspected but their real contribution to bleeding disorders is completely unknown. However, it was shown in human infants that copper deficiency during the pregnancy can result in several structural abnormalities including vascular fragility [12]. Further prospective studies are needed to resolve the important question if trace element deficiencies and/or other nutritional deficiencies might contribute to the picture of subconjunctival bleedings. One interesting case report in a newborn baby has already demonstrated the association of subconjunctival bleedings at birth with maternal malnutrition and vitamin C deficiency in pregnancy [13].

Our data about the appearance and anatomical localization of subconjunctival bleedings were largely similar to those reported by others [4, 8]. The most common appearances were semilunar or macular bleedings immediately outside the limbus. A uniform pattern regarding the position of the bleedings within the eyeball was not recognizable.

A very interesting publication in humans favored the hypothesis that compression of the thorax and abdomen during delivery or forces generated in utero during labor may raise venous pressure to conjunctival vessels and can cause subconjunctival bleedings [8]. In a similar way, every severe venous congestion to the head caused by severe compression of thorax and abdomen was considered to be responsible for subconjunctival bleedings in humans [2, 14]. However, subconjunctival bleedings caused by pressures on the chest or abdomen are often accompanied by further symptoms including facial petechia or ecchymoses [8] that were not seen in our affected calves. Additionally, the unusual high incidence of subconjunctival bleedings of 29.7 per cent within a very short (three month-) examination period reported by Katzman [8] raises doubts whether subconjunctival bleedings in neonatal babies were caused exclusively by compression of the thorax and abdomen during delivery or labor, respectively.

In this context, we have to keep in mind that there are also anatomical differences in the vascular supply of the head between large animals and humans. In animals and humans, the venous blood of the eyeball is mainly drained through the vorticose veins. However, posterior ciliary veins were shown to be constantly present in equine and bovine eyeballs which complement the vorticose and choroidoretinal veins [15]. It is concluded that posterior ciliary veins might play a functional role by providing an additional outflow route for the high volume of choroidal blood in equine and bovine species. These and further anatomical differences make it difficult to compare human and animal studies dealing with the causes for the development of subconjunctival bleedings in newborns.

Our study had several limitations. First of all, the examinations were performed in only one local farm in the south of Germany. Therefore, it is not excluded that specific local conditions such as animal husbandry and feeding might have a major impact to the obtained results. Second, the retrospective nature of this investigation may be responsible for the low incidence and sex predisposition, respectively. Additionally, not all calves included in this study were examined comprehensively by the same veterinarian. There is the probability that calves with subconjunctival bleedings were not identified for different reasons. Further limitations are the lack of complete ophthalmologic examination by a board-certified specialist. For that reason, the simultaneous presence of retinal and subconjunctival bleedings as described previously in foals [4] cannot be excluded, even if the significance and long-term effects have not been proven.

## Conclusion

Subconjunctival bleedings in neonatal calves appear not to be incidental findings. Main causes or associated conditions of subconjunctival bleedings were not found in this case series report.

## Methods

Examinations were performed at the experimental station for Agricultural Sciences of the University of Hohenheim over a six-year period (2015 to 2020). Holstein–Friesian dairy cattle owned by University of Hohenheim were kept in a pen with a separate calving partition. On average 48 calvings per year were recorded. The ratio between male and female calves at birth was slightly biased towards the male sex (Table 3).

**Table 3:** Number of calvings and number of calves with subconjunctival bleedings per year

Year	Female	Male	Total	Cases
2015	20	26	46	3
2016	25	23	48	3
2017	26	29	55	0
2018	20	30	50	0
2019	28	25	53	0
2020	12	25	37	1

At the University of Hohenheim experimental station, all calves (females and males) were examined by experienced veterinary surgeons within 72 h of birth and received a thorough clinical examination according to Good Veterinary Practice (GVP). The evaluation of conjunctival mucous membranes is an important part of GVP. Occurrence and exact localization of subconjunctival bleedings were recorded with the exception of one case (calf 2). Blood samples were taken from only three affected calves after birth due to calving time. Two calves were analyzed hematologically. Hematology was performed using an automated animal blood counter (scil Vet abc Plus + , Scil animal care company, Viernheim, Germany). Blood differential test was manually performed by counting 400 white blood cells on stained blood smears. Serum amounts of iron and/or copper of three calves were determined in external veterinary laboratories (IDEXX, Ludwigsburg, Germany; Biocontrol, Ingelheim, Germany). Reference values were provided by the laboratories or derived from the literature, respectively.

Details of the cow’s and calf’s parturitional and gestational history were recorded. In detail, calf’s age and sex, cow’s gestation time, number of parities, and calving difficulties were noted. Calving difficulty was scored according to Barrier and Haskell [9]: *N* = no assistance; FN = farm assistance; VN = veterinarian assistance; and VC = cesarean section or fetotomy.

Only descriptive statistics were used because of the low number of affected calves.

## Data Availability

All data generated or analysed during this study are included in this published article.
